# Concussion Knowledge, Attitudes and Behaviours Among Australian Taekwondo Athletes: A Cross-Sectional Exploratory Study

**DOI:** 10.3390/sports13110409

**Published:** 2025-11-13

**Authors:** Daniel A. Brown, John Whitting, Zachary Crowley-McHattan, Mike Climstein, Luke Del Vecchio

**Affiliations:** 1 Southern Cross University Combat Research Laboratory, Southern Cross University, Gold Coast 4225, Australia; zac.crowley@scu.edu.au (Z.C.-M.); luke.delvecchio@scu.edu.au (L.D.V.); 2 Faculty of Health, Southern Cross University, Lismore 4280, Australia; john.whitting@scu.edu.au; 3 Faculty of Health, Southern Cross University, Gold Coast 4225, Australia; 4 Physical Activity, Sport, and Exercise Research Theme, Faculty of Health, Southern Cross University, Gold Coast 4225, Australia; 5 Physical Activity, Lifestyle, Ageing and Wellbeing Faculty Research Group, University of Sydney, Sydney 2006, Australia

**Keywords:** sport-related concussion, symptom reporting, combat sports

## Abstract

Background: Sport-related concussion (SRC) is a recognised public health concern, with combat sport athletes particularly vulnerable due to frequent head impacts. In Taekwondo, concussion incidence is comparable to other contact sports, yet underreporting and misconceptions may hinder safe management. Understanding knowledge, attitudes, and behaviours in this cohort is critical for athlete safety. Methods: This cross-sectional online survey was distributed to Australian Taekwondo athletes (AKA). Participants completed demographic, concussion history, and the Rosenbaum Concussion Knowledge and Attitudes Survey. Knowledge (CKI) and attitudes (CAI) indices were derived. Group differences were analysed using non-parametric tests, with associations between knowledge, attitudes, and reporting behaviours explored via correlation and logistic regression. Results: Athletes (*n* = 98) demonstrated good knowledge (mean CKI 19.8/25) and positive attitudes (mean CAI 61.6/75), though misconceptions remained. While 92% indicated they would seek medical attention, 21% reported returning to play the same day as a suspected concussion, and over 20% admitted to concealing symptoms. Higher CAI scores, but not CKI, were associated with safer reporting intentions. Conclusions: AKA showed strong knowledge and attitudes towards concussion; however, risky behaviours persisted. Attitudes, rather than knowledge, were more predictive of reporting behaviours, underscoring the need for interventions that strengthen positive attitudes and cultural support for symptom disclosure.

## 1. Introduction

Sport-related concussion (SRC) is recognised as a major public health issue and remains a priority for athlete safety policy and practice [1,2]. Although SRC occurs across a wide range of sports, combat sport athletes are particularly vulnerable because of the inherent risk of intentional strikes to the head during training and competition [3,4]. The true incidence of concussion in these settings is likely underestimated, as symptoms may be subtle, delayed, or deliberately concealed by the athlete. Research in mixed cohorts of combat sport athletes and coaches has highlighted persistent gaps in the recognition, assessment, and management of concussion [5,6,7], with cultural norms often discouraging athletes from disclosing symptoms or reporting suspected SRC injuries [8].

Taekwondo, a full-contact Olympic combat sport, is characterised by rapid kicking techniques and dynamic sparring, frequently involving head-level strikes capable of producing impact magnitudes associated with concussive injury [9,10,11]. A systematic review reported concussion incidence in Taekwondo competition ranged from 0 to 50 per 1000 athlete exposures (AEs), with a median of 4.9 per 1000 AEs [12]. Similar rates and substantial variability across studies and competition levels have also been identified [13]. This median is comparable to other contact sports such as collegiate ice hockey (2.4 per 1000 AEs) and American football (6.6 per 1000 AEs), but lower than rates reported in mixed martial arts (14.7–28.3 per 1000 AEs) [14,15,16,17,18,19]. Although protective equipment, such as headguards, is mandated in Taekwondo competition, laboratory and field studies have shown that their effectiveness in reducing head acceleration and concussion risk is limited [11,20]. Given the high incidence of head impacts and the likelihood of underreporting, concussion remains an ongoing concern in Taekwondo.

Previous research indicated that many combat sport athletes may fail to recognise concussion symptoms, return to sport prematurely, or actively conceal symptoms from coaches and medical staff [3,5,6,7]. In some studies, more than 40% of professional fighters reported returning to training or competition on the same day as a head injury, while over 20% admitted to concealing symptoms [5]. These behaviours are not explained by knowledge gaps alone [6,21]. They are influenced by the cultural and psychological environment of sport, where resilience and toughness are often prioritised, and where athletes may fear removal from competition or feel pressure from coaches, teammates, or family [5,6,22,23,24,25]. Internal motivators, including love of sport, desire to win, career aspirations, and self-imposed pressure, also play an important role in decisions to return prematurely or conceal symptoms [5].

While knowledge of concussion provides a foundation, it alone may not be sufficient to support safe concussion management. Athletes’ attitudes, beliefs, and levels of trust in coaches and medical professionals are likely to be critical in determining whether concussion symptoms are reported, medical attention is sought and return to play guidelines are followed. Understanding how these factors interact within Taekwondo is essential for designing education and management strategies that are relevant to the sport. Therefore, this study aimed to investigate concussion knowledge, attitudes, and reporting intentions among Australian Taekwondo athletes. The study also examined where Taekwondo athletes received concussion education, their confidence in this education, and how knowledge and attitudes related to behavioural intentions, including seeking medical help and disclosing symptoms.

## 2. Materials and Methods

### 2.1. Study Design

This cross-sectional survey investigated concussion-related knowledge, attitudes, and behaviours among adult Taekwondo athletes in Australia. Data were collected between November 2024 and August 2025 (inclusive) in collaboration with Australian Taekwondo and affiliated clubs. The study received approval from the University Human Research Ethics Committee (HREC: 2024/109), and participation was voluntary and anonymous.

The data-collection period was chosen to align with the Australian Taekwondo competition calendar, capturing both in-season and off-season athletes to maximise participation and representativeness. As an exploratory study, the sample size was determined by practical recruitment feasibility and guided by previous concussion-knowledge studies in combat and contact sports, including Follmer et al. (2020), who surveyed 70 athletes and 35 coaches across mixed martial arts, boxing, kickboxing, and Muay Thai [6]. This approach ensured that the final analytic sample (*n* = 98) provided sufficient representation for the intended non-parametric and exploratory analyses.

### 2.2. Participants

Eligible participants were active Taekwondo practitioners aged 18 years or older, regularly engaged in sparring sessions that involve intentional head-level contact, regardless of formal competition status. Both competitive and recreational athletes were therefore considered routinely exposed to potential head impacts.

### 2.3. Survey

The survey comprised three main sections: demographic information, self-reported concussion history, and concussion knowledge and attitudes assessed via the Rosenbaum Concussion Knowledge and Attitudes Survey (RoCKAS) [26,27]. The survey underwent an initial review for face validity and was pilot tested with a small group of Taekwondo athletes and two Taekwondo coaches to ensure content appropriateness [3]. Feedback was sought on the clarity of items, particularly in relation to ambiguous wording, and the survey was refined on this input.

### 2.4. Demographics

Participants provided details on age, sex, belt rank, years of Taekwondo experience, competition status (recreational or competitive), and highest level of education. These variables were used to characterise the sample and explore potential associations with concussion knowledge and beliefs.

### 2.5. Self-Reported Concussion History

Participants were asked to report whether they had ever been diagnosed with a concussion, if they suspected an undiagnosed concussion, or sustained a concussion within the past 12 months. Participants provided information regarding experiencing a concussion in competition and training environments. Additional items in the survey captured the context of injury (training or competition), the number of concussions, and whether symptoms were disclosed to coaches or medical professionals.

### 2.6. Concussion Knowledge and Attitudes

Participants’ knowledge and attitudes toward concussion were assessed with a modified version of the RoCKAS [27]. Consistent with prior adaptations for non-US settings, minor terminology changes were made to reflect Australian practice. For example, ‘athletic trainer’ was replaced with ‘physiotherapist’ to align with local professional roles [27]. The RoCKAS has been shown to have acceptable internal consistency (Cronbach’s alpha = 0.83), indicating reliable measures of both knowledge and attitudes related to concussion [28]. The RoCKAS is a 55-item survey battery that is broken into five sections. The first two sections consist of ‘true’ and ‘false’ items that assessed factual and applied knowledge, including symptom recognition and misconceptions about concussion. Section 3 presented general opinion statements, while Section 4 included scenario-based items. Both Section 3 and Section 4 used a five-point Likert scale ranging from ‘strongly disagree’ to ‘strongly agree’. The final section focused on symptom recognition, requiring participants to identify common post-concussion symptoms among a list that included distractor items. The RoCKAS scoring framework allowed for two composite scores to be calculated. Section 1, Section 2 and Section 5 constituted the Concussion Knowledge Index (CKI; range 0–25), whereas Section 3 and Section 4 comprise the Concussion Attitudes Index (CAI; range 15–75).

### 2.7. Procedures

The survey was distributed electronically via an online, cloud-based research tool (Qualtrics, Provo, UT, USA). Recruitment was facilitated through Australian Taekwondo’s communication channels, including email invitations, newsletters, and social media posts. Additionally, affiliated Taekwondo clubs across Australia were invited to disseminate the survey to their members. Athletes first accessed an information sheet outlining the purpose of the study, and informed consent was obtained prior to the voluntary completion of the survey. Responses were collected anonymously, and no identifying information was recorded.

### 2.8. Statistical Analyses

Descriptive statistics were used to summarise demographic information, concussion history, and RoCKAS scores. Frequencies and percentages were reported for categorical variables, and means with standard deviations (±SD) or medians with interquartile ranges were reported for continuous variables. Composite scores for the CKI and CAI were calculated according to the RoCKAS scoring protocol [27]. In line with previous research, the item “I would continue playing a sport while also having a headache that resulted from a concussion” was used as a proxy for reporting intention [7,29].

Incomplete responses were handled using pairwise deletion, allowing each analysis to include all available data for the variables under consideration. This approach was selected to retain statistical power while minimising data loss, as several participants provided substantial but incomplete responses. Preliminary checks suggested data were missing at random, supporting the appropriateness of this method.

Because CKI and CAI scores did not meet the assumptions of normality and homogeneity of variance, and several independent variables were ordinal in nature, non-parametric methods were applied to reduce the risk of Type I error. Group comparisons were performed using Mann–Whitney U tests for binary variables such as sex, athlete type, and concussion education.

For variables with “Yes”, “Maybe”, and “No” options, analyses were performed by collapsing “Yes” and “Maybe” into a single category and comparing against “No”. As a sensitivity step, “Maybe” responses were also retained as a separate descriptive category. This allowed us to present the uncertainty reflected in those responses while avoiding very small group sizes that would not support reliable statistical testing.

Associations between CKI or CAI and behavioural intentions were assessed using both group comparisons and correlations. Items coded as “Yes”, “Maybe” or “No” were dichotomised (“Yes/Maybe” versus “No”) for Mann–Whitney U tests and also treated as ordinal for Spearman rank correlations (r_s_). Symptom hiding items with “Never”, “Rarely”, or “Often” options were dichotomised (“Never” versus “Rarely/Often”) for Mann–Whitney U tests, with Spearman correlations conducted as a sensitivity analysis using the original ordinal scale. Exploratory logistic regression analyses were also conducted to examine whether CKI and CAI scores predicted reporting-related behaviours, with age, sex, and athlete type included as covariates. Results are presented as odds ratios with 95% confidence intervals.

Effect sizes for Mann–Whitney U tests were calculated as the rank-biserial correlation, defined as follows:

$$Rrb=\;\frac{2U}{n1n2}-1$$

where *U* = Mann–Whitney U statistic, *n*_1_ = sample size of group 1, *n*_2_ = sample size of group 2.

Interpretation thresholds were ‘very small’ (<0.10), ‘small’ (0.10–0.29), ‘moderate’ (0.30–0.49), and ‘large’ (≥0.50) [30]. For Spearman correlations (r_s_), strength of association was interpreted as ‘negligible’ (0.0–0.10), ‘weak’ (0.1–0.39), ‘moderate’ (0.40–0.69) and ‘strong’ (0.70–0.89) [3]. All tests were two-tailed with significance set a priori at α = 0.05, and analyses were conducted in SPSS (Version 30).

## 3. Results

**Figure 1:**
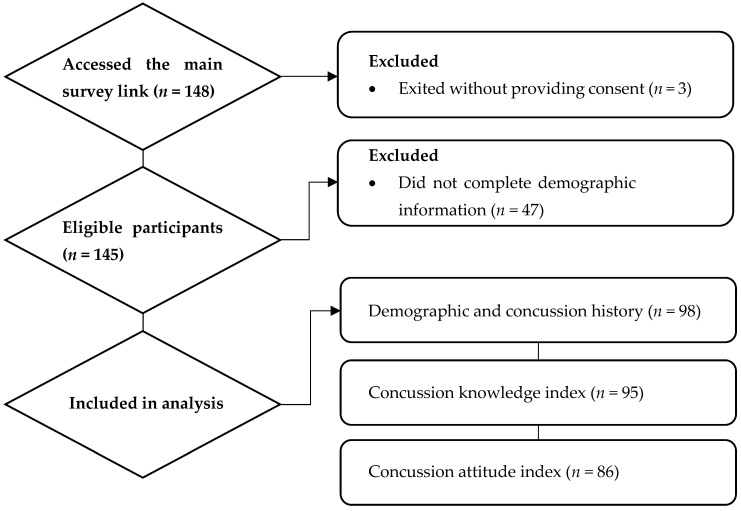
Flow diagram illustrating steps from initial survey access to athletes included in each level of analysis.

A total of 148 athletes accessed the main survey link. Three individuals opted not to provide informed consent and, as per the study protocol, were not able to proceed beyond the consent page. Therefore, we had 145 eligible respondents, of whom 98 completed the demographic, concussion history, and reporting sections; 95 completed the CKI items; and 86 completed the CAI items. Athletes who did not complete the CKI or CAI did not differ significantly from completers by sex, athlete type, or age (*p* > 0.05). A description of the athletes included in the study is displayed in Figure 1.

The mean age of participants was 27.6 years (+12.5), with an average of 12.8 years (+7.2) of Taekwondo experience. Most participants were black belt ranked (92%). Education levels were distributed across undergraduate (34%), high school graduate (33%), postgraduate (15%), certificate/diploma (14%), and less than high school (4%). Sample characteristics, including sex, athlete type, and concussion history, are summarised in Table 1.

### 3.1. Concussion Education

In total, 63% of athletes (n = 62) reported receiving some form of concussion education, most commonly from schools, medical staff, universities, and sport organisations. Less frequently cited sources of concussion education included coaches, family and peers, work, online resources, and first aid courses. Confidence in the quality of education varied, with athletes rating universities, sport organisations, and medical staff most highly, and schools and coaches less favourably (Table 2).

### 3.2. Trust in Medical Professionals and Return-to-Play Behaviours

Most athletes indicated safe intentions regarding concussion management: 92% reported they would seek medical attention, and 87% expressed trust in medical professionals. Nonetheless, 21% reported returning to play on the same day as a suspected concussion, and 22% and 23% acknowledged hiding symptoms from medical staff or coaches, respectively. Internal motivations were the most frequently reported reasons for returning early to training and competition (love of sport 56%, desire to win 39%, self-pressure 31%), while external influences such as coaches (8%) and family (5%) were cited less often (Table 3).

### 3.3. Concussion Knowledge Index

Athletes demonstrated moderate concussion knowledge, with a mean CKI score of 19.8 (+2.7) out of 25. Most participants correctly recognised that concussion symptoms can persist for weeks (99%) and identified common symptoms such as headache, dizziness, and blurred vision (>95%). However, misconceptions were identified. Only 75% recognised that concussion can occur from forces to the body rather than direct head impact, 39% knew that brain imaging is usually normal following concussion, and 18% correctly distinguished concussion from coma. Item-level responses are presented in Supplementary Table S1.

### 3.4. Concussion Attitude Index

Athletes generally reported positive concussion attitudes, with a mean CAI score of 61.6 (+7.6) out of 75 (range 41–74). Most athletes endorsed removal from play following loss of consciousness (93%) and rejected the idea that concussions are less important than other injuries (92%). One quarter of athletes reported they would continue with sport while symptomatic from a concussion. Fewer athletes (60%) agreed that physiotherapists, rather than athletes themselves, should make return to play decisions. Item-level responses are presented in Supplementary Table S2.

### 3.5. Knowledge and Attitudes by Sex, Athlete Type, Concussion Education and History

Exploratory comparisons showed no significant differences in CKI scores by sex, athlete type, concussion education, or concussion history (*p* > 0.05). For CAI, recreational athletes reported more positive attitudes than competitors (U = 471, *p* = 0.021, rrb = 0.33). All other comparisons were non-significant (Table 4).

Associations between CKI and CAI scores and behavioural outcomes are summarised in Table 5. Athletes who indicated they would seek medical attention reported higher CKI (U = 438, *p* = 0.050, rrb = 0.44) and CAI scores (U = 418, *p* = 0.025, rrb = 0.51) than those who would not. Hiding symptoms from medical staff was associated with lower CAI scores (U = 457, *p* = 0.023, rrb = 0.33; r_s_ = −0.24, *p* = 0.026), and a similar trend was observed for hiding symptoms from coaches (U = 526, *p* = 0.052, rrb = −0.27; r_s_ = −0.22, *p* = 0.039). Athletes who expressed an intention to report concussion had significantly higher CAI scores than those who did not (U = 1091, *p* <0.001, rrb = −0.55). CKI scores showed no consistent associations with behavioural outcomes.

Exploratory logistic regression indicated that higher CAI scores were associated with greater odds of intending to seek medical attention (OR = 1.18, 95% CI [1.04, 1.34], *p* = 0.012), and intention to report (OR = 1.16, 95% CI [1.07, 1.25], *p* = 0.001). CKI scores were not significant predictors of this outcome (OR = 1.19, 95% CI [0.95, 1.50], *p* = 0.133). Neither CKI nor CAI predicted trust in medical professionals, concealment of symptoms, or same-day return to play.

## 4. Discussion

The present study examined concussion knowledge, attitudes, and behavioural intentions among Australian Taekwondo athletes. Overall, athletes demonstrated good knowledge and generally positive attitudes, yet misconceptions and risk behaviours were present. Importantly, attitudes rather than knowledge showed stronger associations with safer reporting intentions. These findings highlight the need to look beyond factual knowledge alone and to address the cultural and psychological factors that shape how athletes recognise and respond to concussion.

### 4.1. Concussion Knowledge

The mean CKI score (78%) indicated that Australian Taekwondo athletes possessed a good level of knowledge, comparable to other sporting populations, including Muay Thai (78%), rugby union (76%) and soccer (82%) [7,27,31]. High rates of correct identification of symptoms such as dizziness and headache, acknowledgement that symptoms can persist for several weeks (98%), recognition that the loss of consciousness is not required for diagnosis (97%), and awareness of potential psychological disturbance (95%) reflect an encouraging level of foundational knowledge among Taekwondo athletes. These findings suggest that many athletes were aware of the serious nature of concussion and have retained key safety messages prompted through educational environments.

Despite these encouraging results, several misconceptions remain that may compromise athlete decision-making and contribute to premature return-to-sport. Twenty-five per cent of athletes were unable to recognise that concussion can result from forces applied to the body rather than direct head impact, and 39% understood that brain imaging is typically conducted following concussion [32]. These findings are similar to those reported in competitive Muay Thai athletes, suggesting consistency in knowledge gaps across adult combat sport populations [7]. Such misunderstandings are concerning, as athletes who are cleared of structural injury via imaging may wrongly assume they are also cleared of concussion or may dismiss symptoms if they have not been “knocked out”, potentially disregarding best practice guidelines for return-to-sport [4,32].

More than half of participants reported receiving some form of concussion education, commonly through schools, universities, sport organisations or medical professionals. Confidence in these sources varied, with universities, sport organisations and medical staff rated most highly, where schools and coaches were viewed less favourably. The presence of misconceptions despite exposure to education suggests that existing programmes or methods may lack clarity or consistency, reinforcing the need for tailored, sport-specific initiatives that provide accurate and practical guidance for Taekwondo athletes.

### 4.2. Attitudes and Behaviours

Athletes in this study generally endorsed positive attitudes towards concussion, with mean CAI scores comparable to those reported in competitive Muay Thai (62.7 ± 7.4), suggesting that precautionary attitudes to reporting and management may be a consistent feature across combat sports [7]. Approximately 75% of Taekwondo athletes reported positive intention to disclose concussion, a proportion similar to that of Muay Thai (69%) and soccer (65%) athletes [7,29]. Interestingly, recreational athletes demonstrated more positive attitudes than competitive athletes. Similar trends have been reported in other sports, such as cycling, where competitive athletes displayed less precautionary attitudes [33]. Previous research suggests that competitiveness shapes reporting behaviour, with athletes at higher competitive levels less likely to disclose symptoms due to a focus on performance outcomes and the situational importance of competition [34,35,36]. When viewed in conjunction with existing evidence, these findings suggest that competitive athletes may be more vulnerable to prioritising success over health, highlighting the need for tailored interventions that address barriers unique to high-performance contexts.

Behaviourally, most Taekwondo athletes expressed trust in medical professionals (87%) and reported intention to seek medical attention following a suspected concussion (92%). These proportions are higher than those observed in professional fighters, where almost half indicated low trust in ringside medical providers [5]. Other combat sport cohorts have reported treatment-seeking rates of only 13–26% [3,37], suggesting comparatively favourable care-seeking intentions in this group. However, these were self-reported intentions rather than observed behaviours, and they did not always translate into safe practice. For example, while most athletes reported they would seek medical attention, one in four also indicated they would continue to train or complete despite experiencing concussion symptoms. In addition, 21% of athletes reported returning to training or competition on the same day as a suspected concussion. This proportion is lower than the 51% previously reported in mixed combat sport cohorts [3], yet it still reflects a concerning level of risk. Such variability across studies may reflect contextual or methodological differences, but the consistent pattern is that positive intentions alone do not guarantee safe decisions.

Motivations for unsafe behaviours in this study appear to be driven primarily by internal rather than external pressures. Athletes cited love of sport, desire to win, self-imposed pressure, and career aspirations more frequently than influences from coaches (8%) or family (5%). Overall, 34% of participants reported pressure from themselves, compared with 2% reporting pressure from external sources. This contrasts with previous combat sport research, where only 3.1% reported self-imposed pressure [5]. Prior research across collegiate and combat sports has shown that internal motivations, such as competitiveness, self-imposed expectations, and conformity to cultural norms of masculinity, play a central role in athletes’ decision to continue participation despite symptoms [5,34,38]. External and social pressures, including coach expectations, sport culture, and shared norms that emphasise risk-taking, have been shown to influence athletes’ willingness to disclose concussion symptoms, with lower perceived coach support linked to under-reporting and playing while symptomatic [21,34,38,39]. Together, these findings suggest that concussion reporting is influenced by the interaction between individual motivation and the social environments that reward performance and perseverance.

In contrast to prior work showing that male athletes who conform more strongly to traditional masculine norms are less likely to report concussion symptoms [34] and that female athletes often demonstrate higher reporting intentions in team-sport settings [38], the present study found no significant gender differences in concussion knowledge (CKI) or attitudes (CAI). This may reflect the training structure of Taekwondo, where male and female athletes typically engage in similar sparring routines and coaching environments, potentially minimising gender-specific cultural influences. Furthermore, although higher knowledge scores have been reported among male community football players compared with females, no differences have been reported in attitudes, suggesting that sex-based variation in knowledge does not necessarily influence behavioural intentions [29]. The present findings similarly indicate that, within Taekwondo, sex-based differences in attitudes are unlikely to substantially affect concussion-related behaviour. Nonetheless, future research should continue to examine how gender and other sociocultural factors interact with motivational drivers to shape concussion-disclosure behaviour across sports.

A key finding of this study was that attitudes, rather than knowledge, were more consistently associated with reporting intentions. Athletes with higher CAI scores were more likely to seek medical attention, less likely to conceal symptoms, and scored significantly higher than those who did not intend to report a concussion, reinforcing the link between positive attitudes and safer behavioural intentions. In contrast, CKI scores showed no consistent relationship with behaviour. These results align with previous research demonstrating that increased knowledge does not necessarily translate into safer concussion behaviours or improved reporting [40,41]. This pattern suggests that while educational interventions improve awareness, they must also address the attitudinal and motivational factors that drive behaviour.

### 4.3. Clinical Implication

The findings of this study highlight that although Australian Taekwondo athletes generally demonstrated good concussion knowledge, their attitudes were more strongly linked to reporting behaviours. Previous research has shown that education efforts focused solely on increasing knowledge do not reliably improve reporting intentions among athletes [27,36,40]. Instead, interventions should prioritise strengthening attitudes and addressing the motivations that lead athletes to conceal symptoms [42]. Education that emphasises the consequences of delayed removal from sport, including prolonged recovery, increased days missed, and potential negative effects on performance, may help encourage symptom disclosure and adherence to return to sport guidelines [5,25,37]. In addition, embedding medical staff in training and competition, equipping coaches and high-performance staff with skills in recognition and referral, and creating a supportive concussion reporting culture may strengthen appropriate attitudes and behaviours [21,34,38,43].

### 4.4. Limitations

This study has several limitations. Because the survey was distributed through online channels and club networks, the total number of athletes who received the invitation could not be determined, and a response rate could therefore not be calculated. There is a possibility of self-selection bias, as athletes with greater interest or awareness regarding concussion may have been more likely to participate, potentially inflating estimates of knowledge and attitudes. The sample also represents only a small proportion of active Australian Taekwondo athletes and may not capture the full diversity of states, age groups, or competition levels, which limits generalisability.

The reliance on self-reported data introduces potential recall bias, particularly in relation to concussion history. Social desirability bias may also have influenced responses, as participants could have been inclined to report attitudes or intentions that align with socially accepted views on concussion safety. Although anonymity was emphasised to reduce this effect, it remains possible that some athletes overreported positive intentions or underreported risk behaviours. The decision to combine “Yes” and “Maybe” responses for inferential analyses may have introduced interpretive bias by potentially masking uncertainty between definite and tentative responses. However, this approach was necessary to ensure adequate group sizes for non-parametric testing and to account for the ambiguity frequently seen in self-reported concussion history and behaviours. All response distributions were retained descriptively to maintain transparency. This approach reflects the uncertainty inherent in self-report, although future studies with larger samples may be able to treat this category separately. Finally, multiple group comparisons were conducted using non-parametric tests. While this approach was appropriate for the data, it increases the risk of Type I error. The analyses should therefore be considered exploratory and hypothesis-generating rather than confirmatory.

## 5. Conclusions

Australian Taekwondo athletes demonstrated generally good knowledge of concussion; however, persistent misconceptions and risk behaviours, such as concealing symptoms or returning to play too soon, remain concerning. Attitudes were more strongly associated with reporting behaviour than knowledge, suggesting that educational approaches should not only address factual understanding but also target beliefs, motivations, and decision-making processes surrounding concussion disclosure.

For coaches and sporting organisations, the findings highlight the importance of creating environments where athletes feel supported to report symptoms without fear of losing selection opportunities or competitive standing. Embedding concussion education within regular training, encouraging open dialogue between athletes and medical staff, and ensuring coaches model safe return-to-play practices may strengthen reporting behaviour. Tailored education for combat sport contexts that acknowledge both internal performance pressures and injury safety could further improve outcomes. Collectively, these actions may contribute to safer concussion management and better long-term athlete well-being.

## Figures and Tables

**Table 1:** Athlete characteristics and concussion history (n = 98).

Variable	Category	n	%
Sex	Male	60	61
	Female	38	38
Athlete type	Competitor	71	72
	Recreational	27	28
Concussion diagnosis	Yes	37	38
	Maybe	2	02
	No	59	60
Suspected undiagnosed concussion	Yes	25	26
	Maybe	22	22
	No	51	52
Concussion in the past 12 months	Yes	12	12
	Maybe	10	10
	No	76	78

**Table 2:** Confidence in concussion education by source.

Source	None n (%)	Slight n (%)	Moderate n (%)	Very n (%)	Total n
School	2 (11)	6 (33)	9 (50)	1 (6)	18
Medical staff	0 (0)	3 (25)	7 (58)	2 (17)	12
University	0 (0)	1 (14)	4 (57)	2 (29)	7
Sport organisation	0 (0)	0 (0)	4 (67)	2 (33)	6
Coach	0 (0)	3 (60)	2 (40)	0 (0)	5
Family/friends/peers	0 (0)	0 (0)	3 (100)	0 (0)	3
Work	0 (0)	1 (33)	1 (33)	1 (33)	3
First aid course	1 (33)	1 (33)	1(33)	0 (0)	3
Not recalled	0 (0)	1 (50)	1 (50)	0 (0)	2
Online/journals	0 (0)	0 (0)	2 (100)	0 (0)	2

**Table 3:** Reporting behaviours, motivations and pressure.

Variable	n	%
Reporting behaviours		
Would you seek medical attention if you suspected you had a concussion? (n = 97)	89	92
Trust in medical professionals with regard to concussion (n = 97)	84	87
Hid symptoms from medical staff (Rarely/Often)	22	22
Hid symptoms from coach (Rarely/Often)	23	23
Return to training or competition on the same day as concussion (n = 98)	21	21
Motivations for returning early (n = 96)		
Love of sport	54	56
Desire to win	37	39
Pressure on self	30	31
Career aspirations	17	18
Fear of disappointing the coach	14	15
Feel like there is no choice but to return	11	11
Legacy/reputation	11	11
Lack of knowledge of consequences	8	8
Family	6	6
Money/income	3	3
Endorsements	2	2
None	26	27
Sources of pressure (n = 96)		
Self	23	24
Coach	8	8
Family	5	5
Teammates	4	4
Other	4	4
None	70	73

**Table 4:** CKI and CAI scores by sex, athlete type, concussion history, and education.

Factor	Outcome	Group 1	n1	Mean ± SD	Mdn (IQR)	Group 2	n2	Mean ± SD	Mdn (IQR)	U	*p*	r_b_
Sex	CKI	Male	57	19.7 ± 3.0	21 (19–22)	Female	38	20.1 ± 2.0	21 (19–21)	1086	0.981	0.00
	CAI	Male	52	61.9 ± 7.6	63 (58–68)	Female	34	61.6 ± 7.7	63 (57–66)	911	0.815	0.03
Athlete type	CKI	Comp	71	19.7 ± 2.7	21 (19–21)	Rec	24	20.4 ± 2.5	21.0 (19–22)	664	0.100	0.22
	CAI	Comp	64	60.7 ± 7.9	63 (57–66)	Rec	22	65.0 ± 5.8	67 (61–69)	471	0.021	−0.33
Diagnosed concussion	CKI	Yes	38	19.8 ± 2.6	20 (19–21)	No	57	19.9 ± 2.7	21 (19–21)	1015	0.598	−0.06
	CAI	Yes	36	61.4 ± 7.9	63 (58–68)	No	50	62.1 ± 7.5	64 (58–68)	840	0.599	−0.07
Suspected	CKI	Yes	45	19.8 ± 2.4	21 (19–21)	No	50	19.9 ± 2.9	21 (19–21)	1045	0.542	0.07
	CAI	Yes	41	61.2 ± 8.5	63 (57–67)	No	45	62.4 ± 6.8	64 (58–68)	875	0.681	−0.05
Concussion in the past 12 months	CKI	Yes ^	21	19.4 ± 3.0	20 (17–22)	No *	74	20.0 ± 2.6	21 (19–21)	700	0.483	−0.10
	CAI	Yes ^	20	59.2 ± 9.3	62 (50–66)	No *	66	62.6 ± 6.9	64 (59–68)	524	0.162	−0.21
Education	CKI	Yes	60	20.0 ± 2.7	21 (19–22)	No	35	19.5 ± 2.6	20 (18–21)	1197	0.248	0.14
	CAI	Yes	54	62.0 ± 7.7	64 (57–68)	No	32	61.5 ± 7.6	63 (58–68)	903	0.730	0.05

Abbreviations: CAI, concussion attitude index; CKI, concussion knowledge index; Comp, competitor; IQR, interquartile range; Mdn, Median; Rec, recreational; SD, Standard deviation. Note: Yes are “Yes and Maybe” responses. ^ represents athletes who reported a concussion in the past 12 months. * represents athletes who did not report a concussion in the past 12 months.

**Table 5:** Associations between concussion knowledge, attitudes, and behaviours.

Behaviour	Outcome	Yes (n, Mdn (IQR))	No (n, Mdn (IQR))	U	*p*	r_b_	r_s_ (*p*)
Seek medical attention	CKI	87, 21 (19–21)	7, 18 (16–20)	438	0.050	0.44	-
	CAI	79, 63 (58–68)	7, 57 (48–64)	418	0.025	0.51	-
Trust medical professionals	CKI	82, 21 (19–21)	12, 20 (16–20)	625	0.124	0.27	-
	CAI	74, 64 (59–68)	12, 58 (49–65)	592	0.065	0.33	-
Intention to report	CKI	22, 20 (18–71)	64, 21 (19–22)	838	0.175	−0.19	-
	CAI	22, 57 (49–63)	64, 64 (60–68)	1091	<0.001	−0.55	-
Same-day return to play	CKI	19, 20 (19–21)	67, 21 (19–22)	607	0.182	0.05	-
	CAI	19, 58 (50–68)	67, 64 (59–68)	499	0.152	0.22	-
Hid symptoms (medical staff)	CKI	21, 20 (18–22)	73, 21 (19–21)	723	0.468	−0.06	−0.095 (0.359)
	CAI	21, 58 (50–65)	65, 64 (59–68)	457	0.023	−0.33	−0.240 (0.026)
Hid symptoms (coach)	CKI	23, 20 (16–22)	71, 21 (19–21)	668	0.155	−0.18	−0.173 (0.093)
	CAI	23, 61 (50–66)	63, 64 (59–68)	526	0.052	−0.27	−0.223 (0.039)

Abbreviations: CAI, concussion attitude index; CKI, concussion knowledge index; IQR, interquartile range; Mdn, median. Note: Yes are “Yes and Maybe” responses.

## Data Availability

The data presented in this study are not publicly available due to ethical restrictions and participant confidentiality, as outlined in the study’s ethics approval. De-identified data may be made available from the corresponding author upon reasonable request.

## Supplementary Materials

The following supporting information can be downloaded at: https://www.mdpi.com/article/10.3390/sports13110409/s1, Table S1: Concussion Knowledge Index item responses (n = 95); Table S2: Concussion attitude index item response (n = 86).

## Abbreviations

The following abbreviations are used in this manuscript:

AE	Athlete Exposures
CAI	Concussion Attitudes Index
CI	Confidence interval
CKI	Concussion Knowledge Index
HREC	Human Research Ethics Committee
IQR	Interquartile range
mTBI	Mild traumatic brain injury
OR	Odds ratio
RoCKAS	Rosenbaum Concussion Knowledge and Attitudes Survey
SD	Standard deviation
SRC	Sport-related concussion
