# Supplementary material for: Concussion Knowledge, Attitudes and Behaviours Among Australian Taekwondo Athletes: A Cross-Sectional Exploratory Study
Source: Sports (Basel). 2025 Nov 13;13(11):409. doi: 10.3390/sports13110409 (PMC12656089; doi:10.3390/sports13110409)
Supplement: Supplementary file 1 [file sports-13-00409-s001.zip › sports-3936413-supplementary.pdf]

Supplementary Table S1: Concussion Knowledge Index item responses (n = 95).

Item	Correct (n)	Correct (%)
**Section 1**		
1. There is a possible risk of death if a second concussion occurs before the first one has healed	77	81
3. People who have had one concussion are more likely to have another concussion	72	76
5. In order to be diagnosed with a concussion, you have to be knocked out	92	97
6. A concussion can only occur if there is a direct hit to the head	71	75
7. Being knocked unconscious always causes permanent damage to the brain	66	69
8. Symptoms of a concussion can last for several weeks	93	98
9. Sometimes, a second concussion can help a person remember things that were forgotten after the first concussion	88	93
11. After a concussion occurs, brain imaging (e.g., CAT Scan, MRI, X-Ray, etc.) typically shows visible physical damage (e.g., bruise, blood clot) to the brain	37	39
12. If you receive one concussion and you have never had a concussion before, you will become less intelligent	87	92
13. After 10 days (about 1 and a half weeks), the symptoms of a concussion are usually completely gone	52	55
14. After a concussion, people can forget who they are and not recognise others, but be perfect in every other way	37	39
16. Concussions can sometimes lead to emotional disruptions	90	95
17. An athlete who gets knocked out after getting a concussion is experiencing a coma	17	18
18. There is rarely a risk to long-term health and well-being from multiple concussions	74	78
**Section 2**		
1. It is likely that Player Q's concussion will affect his long-term health and well-being	65	68
2. It is likely that Player X's concussion will affect his long-term health and well-being	84	88
3. Even though Player F is still experiencing the effects of the concussion, her performance will be the same as it would have been had she not suffered a concussion	90	95
**Section 5**		
Amnesia	86	91
Blurred vision	91	96
Confusion	89	94
Dizziness	91	96
Headache	92	97
Loss of consciousness	78	82
Nausea	86	91
Sleep disturbances	80	84

Abbreviations: CAT, computerised tomography; MRI, magnetic resonance imaging.

Supplementary Table S2: Concussion attitude index item response (n = 86).

Item	Correct (n)	Correct (%)
**Section 3**		
1. I would continue playing a sport while also having a headache that resulted from a concussion	65	75
2. I feel that coaches need to be extremely cautious when determining whether an athlete should return to play	74	86
5. I feel that concussions are less important than other injuries	79	92
6. I feel that an athlete has a responsibility to return to a game, even if it means playing while still experiencing symptoms of a concussion	75	87
7. I feel that an athlete who is knocked unconscious should be taken to the emergency room	80	93
**Section 4**		
1. I feel that Coach A made the right decision to keep Player R out of the game	78	91
2. Most athletes would feel that Coach A made the right decision to keep Player R out of the game	59	69
3. I feel that Athlete M should have returned to play during the first game of the season	74	86
4. Most athletes would feel that Athlete M should have returned to play during the first game of the season	58	67
5. I feel that Athlete O should have returned to play during the semifinal playoff game	70	81
6. Most athletes feel that Athlete O should have returned to play during the semifinal playoff game	50	58
7. I feel that the physiotherapist, rather than Athlete R, should make the decision about returning Athlete R to play	52	60
8. Most athletes would feel that the physiotherapist, rather than Athlete R, should decide about returning Athlete R to play	45	52
9. I feel that Athlete H should tell his coach about the symptoms	75	87
10. Most athletes would feel that Athlete H should tell his coach about the symptoms	57	66
